# A genomics perspective of personalized prevention and management of obesity

**DOI:** 10.1186/s40246-024-00570-3

**Published:** 2024-01-29

**Authors:** Kalliopi K. Gkouskou, Maria G. Grammatikopoulou, Evgenia Lazou, Theodora Vasilogiannakopoulou, Despina Sanoudou, Aristides G. Eliopoulos

**Affiliations:** 1 Department of Biology, Medical School, National and Kapodistrian University of Athens, Mikras Asias 75, 11527 Athens, Greece; 2 GENOSOPHY P.C., Athens, Greece; 3 Unit of Immunonutrition and Clinical Nutrition, Department of Rheumatology and Clinical Immunology, University General Hospital of Larissa, Faculty of Medicine, School of Health Sciences, University of Thessaly, Larissa, Greece; 4 Clinical Genomics and Pharmacogenomics Unit, 4th Department of Internal Medicine, Medical School, National and Kapodistrian University of Athens, Athens, Greece; 5 Center for New Biotechnologies and Precision Medicine, Medical School, National and Kapodistrian University of Athens, Athens, Greece; 6 Biomedical Research Foundation of the Academy of Athens, Athens, Greece

**Keywords:** Obesity, Nutrigenetics, Precision nutrition, Precision medicine, Nutrients, Exercise, Chrononutrition, GRS, PRS, SNP

## Abstract

This review discusses the landscape of personalized prevention and management of obesity from a nutrigenetics perspective. Focusing on macronutrient tailoring, we discuss the impact of genetic variation on responses to carbohydrate, lipid, protein, and fiber consumption. Our bioinformatic analysis of genomic variants guiding macronutrient intake revealed enrichment of pathways associated with circadian rhythm, melatonin metabolism, cholesterol and lipoprotein remodeling and PPAR signaling as potential targets of macronutrients for the management of obesity in relevant genetic backgrounds. Notably, our data-based in silico predictions suggest the potential of repurposing the SYK inhibitor fostamatinib for obesity treatment in relevant genetic profiles. In addition to dietary considerations, we address genetic variations guiding lifestyle changes in weight management, including exercise and chrononutrition. Finally, we emphasize the need for a refined understanding and expanded research into the complex genetic landscape underlying obesity and its management.

**Supplementary Information:**

The online version contains supplementary material available at 10.1186/s40246-024-00570-3.

## Introduction

### The genetic background of obesity

Obesity is a major global health concern, and its prevalence is steadily increasing worldwide. If the current trajectory persists, it is projected that by the year 2025, approximately one billion adults, corresponding to nearly 20% of the global population, will be living with obesity [1].

Changes in food availability and consumption have been major drivers of the rapid increase in overweight and obesity. However, genetics has emerged as the key parameter predisposing individuals to high adiposity in an obesogenic environment. Twin, family, and adoption studies have estimated the heritability of obesity to be between 40 and 77% [2]. Notably, thinness is also a heritable trait [3].

Within this context, genome-wide association studies (GWASs) have provided valuable insights into genetic variants associated with body mass index (BMI), a proxy for obesity, as well as other adiposity traits, such as the waist-to-hip ratio, which is a proxy for body fat distribution [1, 4, 5]. Technological developments have further enabled the identification of new genetic loci associated with thinness or obesity. For example, whole-exome sequencing of 640,000 individuals has recently identified several rare gene variants involved in protection from obesity [6]. These discoveries have led to the development of genetic risk scores (GRSs) comprising tens or hundreds of ‘at-risk’ genetic variants as susceptibility tools for overweight and obesity [7, 8]. Furthermore, polygenic risk scores (PRSs), which represent the weighted sum of thousands or millions of trait-associated alleles from GWASs, have been developed as a single quantitative measure of inherited susceptibility. For example, a PRS developed by Khera et al. [9] enables the stratification of individuals of European ancestry based on their risk of developing obesity. Although this PRS was associated with minimal differences in body weight at birth, it predicted differences in body weight trajectories and the risk of developing severe obesity in adolescence [9].

GWASs have also highlighted biological processes and pathways that may impact adiposity. Of particular interest is the observation that the majority of single nucleotide polymorphisms (SNPs) associated with obesity is linked to genes expressed in the central nervous system (CNS) rather than the endocrine system or adipose tissue [10]. This finding likely reflects the central role played by hypothalamic circuits in regulating fundamental aspects of energy regulation, including food intake, taste, and behavioral aspects of eating. Early-onset adiposity in monogenic forms of obesity has been causally linked to loss-of-function mutations in genes controlling appetite, including the melanocortin 4 receptor (MC4R) [11]. Other studies have shown that dietary habits and behavioral eating have a strong genetic influence, with 83 out of 85 curated dietary habits found to be significantly heritable and associated with different SNPs [12].

Interestingly, the brain and the CNS are also primary targets of biomolecules associated with aging [13]. While the existing parallels between excess adiposity and the aging process, also referred to as ‘adipaging’, have primarily been attributed to dysfunctional adipose tissue, obesity has also been associated with adverse changes in brain function and structure [14]. Genetic factors may link obesity with accelerated brain aging. For example, the SNPs rs7412 and rs429358 in exon IV of the apolipoprotein E gene generate the APOE4 variant, which is associated with elevated levels of circulating and tissue cholesterol, oxidized low-density lipoprotein (LDL), and increased risk for late-onset Alzheimer's disease (reviewed previously in [15]).

Unraveling the mechanisms and biological pathways that underlie adiposity traits could thus uncover new targets and strategies to tackle obesity and decelerate aging, as well as other comorbidities. In this regard, it is important to gain insights into the impact of lifestyle within the context of genetic predisposition. Nutrigenetics, a clade of Genetics, explores the role of genetic variation in individual responses to dietary components, offering tremendous potential for the personalized nutritional management of obesity and other diet-related pathologies.

### Bottlenecks in reducing the burden of obesity

Currently, a large number of different nutritional and other lifestyle approaches have been proposed in an attempt to achieve healthy weight. Different hypocaloric diets aimed at producing a negative energy balance and substantial weight loss are applied [16], but macronutrient recommendations vary widely among different professional societies [17]. These ‘one-size-fits-all’ approaches do not account for the diverse responses that occur due to the inherent heterogeneity of obesity [18]. As demonstrated by the POUNDS Lost study [19], these regimens exhibit significant variability in terms of their effectiveness in achieving weight loss, highlighting the inadequacy of current strategies in delivering comprehensive and personalized care to individuals suffering from obesity.

Recently, the contribution of individual macronutrients to promoting weight loss in people with obesity has received increasing attention. The use of different macronutrient ratios may overcome the metabolic difficulties associated with the consumption of specific macronutrients (i.e., carbohydrates) and correct for the poor compliance associated with very-low-calorie diets. Furthermore, a plethora of research suggests that “not all calories are equal” and that different macronutrients may promote weight loss in a more efficient manner [20, 21].

Although nutritionists and dieticians are aware of the energy that each macronutrient may yield, the exact role of each macronutrient and its subtypes in weight loss remain under investigation. Different protein sources (vegetable versus animal), specific sugars (simple or complex), amino acids and fatty acids [saturated (SFA), monounsaturated (MUFA), polyunsaturated (PUFA), or trans] are metabolized differently depending on one’s unique genetic background [22].

Here, we review the research evidence supporting the interplay between macronutrient intake, lifestyle and genetic background within the framework of weight management in individuals with obesity (Additional file 1: Table S1). We also explored how personalized, genotype‐based knowledge could inform recommendations for the dietary management of obesity, aiming at effective weight loss and healthy body weight maintenance.

The present review primarily addresses the use of GRSs and PRSs rather than individual SNPs because the former offer a more comprehensive approach to the management of obesity by combining multiple SNPs linked to BMI. Additionally, we further discuss SNPs that exhibit strong associations with weight management and are thought to be involved in dietary or physical activity (PA) pathways.

## Personalized macronutrient consumption for body weight loss based on genetic profile

### Carbohydrate tailoring

The role of carbohydrates in weight gain and obesity remains a subject of ongoing debate, highlighting the absence of a universal solution for diet and weight management. When considering the proportion of carbohydrates in total dietary energy, randomized trials suggest a small advantage of lower carbohydrate intake over low-fat diets for weight loss. However, these trials exhibit significant heterogeneity in terms of caloric restriction strategies and intensity, making it challenging to draw definitive conclusions about the efficacy of reducing total carbohydrate intake by itself. In contrast, more consistent evidence supports the significance of carbohydrate sources and quality in weight management. Additionally, significant attention has been given to foods with a high glycemic load, which includes rapidly digestible carbohydrates such as refined grains, potato products, and added sugars [23]. Ongoing research in this domain suggests that high-glycemic-load diets have the potential to elevate insulin secretion and shift the allocation of substrates toward fat storage, ultimately contributing to weight gain [24, 25].

This clinical research supports the need for the development of precision nutritional strategies, as significant heterogeneity in individual glucose responses to dietary interventions has been documented. For example, two independent clinical studies reported that individuals with high baseline insulin levels achieve greater weight loss when adhering to low-glycemic-load diets than when they are with low baseline insulin levels [26, 27]. In this regard, it has recently been reported that genetic variants associated with glucose-stimulated insulin secretion play important roles in this carbohydrate–insulin interplay. A genetic predisposition to higher levels of glucose-stimulated insulin secretion also predicted higher adult BMI. Because the use of insulin in combination with oral glucose tolerance testing may not always be feasible, it has been proposed that genetic testing for insulin secretion-associated variants could be highly valuable [28].

Several studies have demonstrated a clear relationship between genetic susceptibility to obesity and carbohydrate intake and weight gain, especially when carbohydrates are refined. For example, Qi et al. [29] used a GRS based on 32 SNPs to stratify individuals according to their genetic predisposition to obesity. They found that individuals with high GRSs were more susceptible to increased BMI when consuming sugar-sweetened beverages than were those with low GRSs for obesity [29]. Subsequent studies have confirmed these associations [30, 31]. Notably, unlike GRSs, which encompass tens of genetic variants, the significance of single SNPs in modifying body weight in relation to carbohydrate intake has been less evident [32–34].

Variability has also been observed in individual responses to carbohydrate digestion. Before they enter the bloodstream, carbohydrates must undergo a process of digestion initiated by the action of salivary α-amylase. Higher salivary amylase levels accelerate starch breakdown, elevating blood glucose concentrations faster following starch digestion [35]. The levels and activity of salivary amylase differ among individuals, and this difference is partly determined by copy number variations (CNVs) in the salivary α-amylase gene (*AMY1*). In some populations, low *AMY1* CNV is associated with high BMI to varying degrees [36–40], but this association does not appear to occur in other populations [41, 42] (Genosophy, unpublished data). These discrepancies may be methodological [41] or due to variations in starch intake between individuals [43]. In this context, a GRS was developed using nine *AMY1* SNPs, where a higher *AMY1*-GRS indicated greater salivary amylase activity. High carbohydrate intake significantly influenced the association between the *AMY1*-GRS score and changes in BMI and waist circumference. Specifically, individuals with a greater *AMY1*-GRS (i.e., higher amylase activity) experienced more pronounced increases in adiposity when their dietary carbohydrate intake was high but showed less adiposity gain when their carbohydrate intake was low [44]. The microbiota also contributes to the distinct way in which individuals metabolize carbohydrates and the varying impact of carbohydrates on one’s physiology. The CNV of *AMY1* has been shown to influence the diversity and function of the human oral and gut microbiomes. After following a fixed diet, individuals with low *AMY1* CNV exhibited an abundance of microbes with an enhanced capacity for complex carbohydrate breakdown, whereas those with high *AMY1* CNV were enriched in microbiota linked to nondigestible starch fermentation. Fermentation of ‘resistant’ starch resulted in greater concentrations of fecal short-chain fatty acids (SCFAs), which was experimentally linked to greater adiposity [45].

Thus, genetic data should be taken into consideration when tailoring carbohydrate consumption for weight loss.

### Tailoring lipid consumption

Lipids constitute a remarkably varied array of molecules, each endowed with specific biological roles. They function as a source of energy, contribute to the structure and operation of cell membranes, serve as precursors for signaling molecules, and play a crucial role in facilitating the absorption of fat-soluble vitamins and essential nutrients. This inherent functional diversity of lipids may account for the inconclusive findings in studies related to weight management and overall fat consumption [46, 47].

The Dietary Guidelines for Americans formerly recommended that less than 30% of total energy intake be derived from fat [48], although this guidance has more recently been moderated to a range of 25–35% [49]. This adjustment may have been influenced by the recognized benefits of the Mediterranean diet, which is characteristically rich in fats [50, 51]. In Europe, recommendations for dietary fat intake exhibit even greater variability, spanning from 15 to 40% of total energy [17]. This finding underscores the complex roles of fats in health and metabolism, emphasizing the necessity of tailoring dietary fat intake based on individual genetic profiles.

Thus far, a limited number of studies have investigated the genetic risk scores of obesity in relation to fat intake and body weight regulation. In one study [52], a GRS comprising 63 obesity-related SNPs was used to stratify individuals according to their genetic susceptibility to obesity. A high GRS was associated with an elevated BMI in people consuming a diet rich in saturated fat. Similarly, a cross-sectional study involving 48,170 Caucasian European adults and employing a GRS based on 93 SNPs, lower levels of total fat consumption and saturated fat in particular, could mitigate the strength of the association between genetic predisposition to obesity and BMI [53]. Both studies pointed to the health benefits of reducing saturated fat intake overall; however, this is even more important for individuals who are genetically susceptible to high adiposity. In another study using a GRS incorporating 77 SNPs related to obesity, a diet rich in long-chain n–3 PUFAs and fish mitigated the effects of genetic predisposition to long-term weight gain [54].

Evidence also suggests that lean body mass (LBM) may play a part in governing appetite and energy intake, thereby influencing body adiposity. In a recent study examining the impact of five variants linked to LBM, individuals with overweight and obesity who exhibited a greater genetic predisposition to higher LBM experienced more substantial benefits from a low-fat weight-loss diet, particularly in terms of improvements in appetite and body adiposity [55].

Earlier research has also provided links between single SNPs and the response to the amount of fat consumed. Based on specific polymorphisms, some people may achieve greater weight loss when adhering to high-fat diets [56, 57], while others may respond better to diets with a low fat content [58–62]. Other studies addressing the quality of fat have shown that saturated fat can increase the risk of obesity in people carrying specific variants, whereas the consumption of MUFAs and PUFAs can have the opposite effect on people at risk [63, 64]. Therefore, in addition to quantity, the quality of fat consumed may influence the genetic predisposition to obesity in a manner that may lead to different body weight outcomes.

The specific biological mechanisms underlying these effects are still being explored. Ongoing research indicates that various forms of dietary fat may induce epigenetic changes that could differ among genotypes. This divergence may result in distinct gene expression patterns and the potential dysregulation of specific metabolic pathways [65]. One such example is the common genetic variant − 265 T > C (rs5082) within the promoter of the apolipoprotein A-II gene (*APOA2*). *APOA2* CC homozygotes at − 265 T > C are at increased risk of obesity when consuming a diet high in SFA compared with TT carriers, but they have no increased risk of obesity when consuming a low-SFA diet [66–68]. This diet-dependent outcome related to obesity may be explained by the methylation status of the CC *APOA2* promoter, which leads to reduced *APOA2* expression in people consuming a high-SFA diet [69]. In turn, this has been linked to the dysregulation of metabolic pathways implicated in obesity and food intake [69]. These findings identify potential mechanisms by which saturated fat influences obesity risk and offer new insights into ongoing investigations of the relationship between fat intake and human health.

As lipids significantly contribute to the taste and texture of food, high-fat foods can provide an exceptionally rewarding eating experience, especially for individuals genetically predisposed to obesity, who may be more susceptible to finding pleasure and reward in food [12]. Dietary replacement of SFAs with MUFAs/PUFAs in commonly consumed foods such as milk and dairy could be a good strategy to attenuate obesity in people who are genetically at risk. This approach has already been proven to be beneficial for individuals with moderate cardiovascular risk [70]. We propose that these foods maintain their palatability and taste to ensure that they are appealing and successful choices for people genetically predisposed to obesity.

### Personalizing protein consumption for body weight management according to genetic background

High-protein diets have gained increased popularity as promising strategies for regulating body weight. Mechanisms that may account for weight loss following high-protein diets include 1) increased secretion of satiety hormones, 2) reduced secretion of orexigenic hormones, 3) improvement of glucose homeostasis and 4) elevated thermic effects of foods rich in protein, suggesting that the body expends more calories during the processes of digesting, absorbing, and utilizing dietary protein than during the process of accessing other macronutrients [71, 72]. However, there are potential concerns when opting for a high-protein diet. For example, when the energy demand is low, excess protein intake will be converted to glucose or ketone bodies that contribute to an undesirable positive energy balance, especially if weight loss is the goal [73]. The source of protein is also an important parameter because excessive consumption of animal-based protein may be accompanied by elevated saturated fat, which increases the risk of heart disease and hyperlipidemia [71] and counteracts weight loss efforts [74, 75].

Several lines of evidence suggest that protein intake may have different effects on body weight depending on the genetic background, providing options for personalized dietary interventions. For example, individuals with a high GRS for obesity are more susceptible to high adiposity when consuming proteins of animal origin protein [52, 76], while the opposite trend is observed when a diet rich in plant-based protein is followed [77].

In a recent study, several SNPs were shown to be associated with favorable responses to a moderately high-protein diet (30% energy from proteins), including improved reductions in waist circumference and total body fat [78]. Two fat mass and obesity associated (*FTO)* gene locus variants were among these SNPs, confirming the findings of previous intervention studies demonstrating that a hypocaloric diet combined with increased protein intake alleviates the influence of *FTO* variants on obesity [79, 80]. The exact mechanisms by which dietary protein modifies the effects of *FTO* on adiposity are not fully understood. One possible explanation is the control of appetite and food cravings in hypocaloric diets, which is known to be influenced by *FTO* genotype [81].

In contrast, however, a meta-analysis conducted in 2014 failed to establish a clear link between protein intake, *FTO* genetic variants and obesity [82]. This conundrum could be explained by the fact that overall caloric intake was not taken into consideration in all relevant observational studies. A key aspect of *FTO*-protein axis outcomes is that high-protein diets must be combined with hypocaloric diets for effective body weight control in genetically susceptible individuals. In high-calorie diets, high protein intake may result in mitigation or even the reverse effect of protein on body weight in adults and children carrying the *FTO* risk allele [83].

In addition to *FTO*, other obesity polymorphisms have been explored for their ability to regulate protein levels in individuals fed hypocaloric diets. These polymorphisms include *MC4R* [84], brain-derived neurotrophic factor (*BDNF*) [85] and the transcription factor AP-2 beta (*TFAP2B*) [86], all of which are associated with improved responses to hypocaloric, low-protein diets. Considering the aforementioned caveats of high-protein consumption in body weight management, genetics could offer opportunities for optimizing benefits while minimizing potential drawbacks.

### Tailoring fiber consumption

High-fiber intake can predict weight loss, particularly when combined with an overall reduction in caloric intake [87, 88]. One possible mechanism accounting for this benefit is that dietary fiber may limit the absorption of dietary fats in the gut [89]. Only a limited number of studies have been conducted on the influence of genetic variants and fiber intake on obesity incidence. In one of them [90], a PRS comprising 2,100,302 SNPs [9] was used to stratify 3098 children and adolescents according to their genetic susceptibility to obesity. Individuals with high PRSs were shown to attenuate their risk when consuming a diet rich in fiber. Another study investigated the combined effect of variations in *FTO* and adrenoceptor beta 2 (*ADRB2*), a gene encoding the beta-2 adrenergic receptor that is implicated in lipolysis and energy release [91, 92]. Individuals at high risk for obesity significantly reduce waist circumference, fat mass and percent body fat when they consume a diet high in fiber [93]. Similar findings were made for a risk score comprising multiple *FTO* SNPs (rs1121980, rs1421085, rs9939973, rs8050136, rs17817449, and rs3751812) [94], overall complementing the reported value of *FTO* variants in personalized hypocaloric, plant-based, high-protein diets for improved weight management [79, 80].

Dietary fiber consumption has also been linked to improved metabolic health and a reduced risk of developing various obesity-related conditions, including type 2 diabetes [95, 96]. An interesting finding regarding genetic variation and fiber intake relates to the SNP rs7903146 *TCF7L2* (transcription factor 7 like 2), which is known to yield the highest risk of type 2 diabetes (T2D) in Caucasians to date [97, 98]. *TCF7L2* encodes a transcription factor that is a member of the Wnt signaling pathway in pancreatic islet β-cells and other cell lineages and glucose-metabolizing tissues, including the liver. Increased fiber intake has been associated with protection from T2D but only among carriers of the nonrisk allele C of the *TCF7L2* SNP rs7903146 [99]. In line with this association, improved weight loss upon fiber consumption (> 25 g/day) is achieved only among individuals homozygous for the allele C of the *TCF7L2* SNP rs7903146 and is not detected in *TCF7L2*-T risk allele carriers [100]. Although these findings cannot be translated into dietary advice for carriers of the *TCF7L2* risk allele, they show that fiber-rich diets are protective against T2D in all individuals carrying nonrisk alleles and thus are incorporated into personalized dietary recommendations.

### Pathways and mechanisms predicted to relate to actionable genomic variants for macronutrient stratification

To gain insight into the pathways and mechanisms that may underpin the aforementioned macronutrient–SNP interactions in obesity management, we performed functional enrichment analysis using the bioinformatics web interface Flame v 2.0 [101]. Gene names associated with 313 SNPs reported to guide macronutrient intake (carbs, fat, protein and fiber; Additional file 1: Table S2) were processed through FLAME to identify the respective genes, thus generating the Actionable Gene List for Obesity Management (AnGeL; Additional file 1: Table S2). Reassuringly, by analyzing this gene list against the DISGENET database and plotting the results as an interaction network, several nutrition-related disease ontologies were identified (Additional file 2: Figure S1). The risk factors included obesity, T2D, coronary artery disease and irregular blood pressure (Additional file 1: Table S3).

**Fig. 1:**
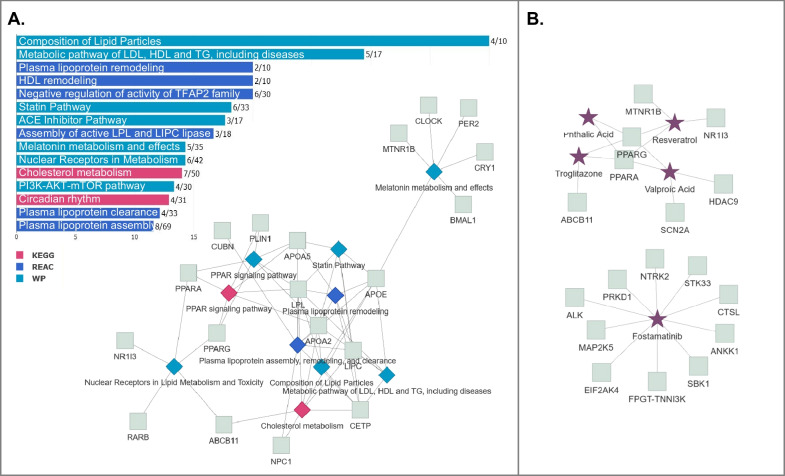
Pathways and therapies predicted to underpin the effects of genetic variants on macronutrient responses in individuals with obesity. **A** The gene names associated with 313 SNPs reported to guide macronutrient intake (carbs, fat, protein and fiber; Additional file 1: Table S2) were analyzed against KEGG, WikiPathways and REACTOME (REAC) combined through the FLAME interface (https://bib.fleming.gr:8084/app/flame). The 15 most enriched pathways (based on − log10 *P* values) from these databases are shown in the bar chart, and 10 of them were plotted as pathways versus genes to show their interconnections. **B** The gene list was analyzed against the DRUGBANK database (https://go.drugbank.com/) through FLAME. Drugs and other therapies (purple stars) predicted to mostly associate with this gene set are plotted in relation to target genes

Τo interpret AnGeL as biological functions and pathways, we combined KEGG, Wiki and REACTOME pathway enrichment analyses using the WebGestalt application tool [102], and the results were processed through FLAME. The most enriched pathways were related to circadian rhythm and melatonin metabolism, cholesterol and lipoprotein remodeling, and the peroxisome proliferator-activated receptor (PPAR) signaling pathway (Fig. 1A), all of which have been implicated in excess adiposity, obesity-related hypertension and diabetes. It is therefore envisaged that these pathways may be prime targets of macronutrients for the management of obesity in relevant genetic backgrounds.

Further enrichment analysis of AnGeL was conducted against the DRUGBANK database and revealed a significant association with fostamatinib (Fig. 1B), an orally administered SYK inhibitor approved for the treatment of chronic immune thrombocytopenia (Additional file 1: Table S3). We note that among the ten fostamatinib gene targets in AnGeL, MAP2K5, ALK, EIF2AK4, NTRK2 and STK33 have been linked to the extracellular-signal-regulated kinase (ERK) mitogen activated protein kinase (MAPK) signaling pathway. SYK inhibitors are known to suppress TPL2 [103], a MAP3 kinase with a pivotal role in inflammatory ERK activation [104]. Interestingly, TPL2 ablation in mice results in improved lipid and glucose balance, enhanced insulin sensitivity, and reduced inflammation in response to a high-fat diet [105], thus suggesting potential mechanistic connections between intervention diets and insulin sensitivity in the context of obesity management. Additionally, fostamatinib has demonstrated effectiveness in ameliorating autoimmune diabetes in mouse models [106]. Consequently, we propose that further investigations be conducted to explore the potential of fostamatinib for the treatment of obesity focusing on relevant genetic profiles.

The aforementioned DRUGBANK enrichment analysis also indicated that the PPAR pathway is a “drugable” target in the context of obesity and its comorbidities (Additional file 1: Table S3). Potential options for intervention may include troglitazone, a potent PPAR-γ agonist utilized in the management of type 2 diabetes, and resveratrol, a naturally occurring polyphenol found in grapes, peanuts, blueberries, rhubarb, and wine that has been associated with the so-called “French paradox”. This refers to the relatively low rates of obesity and CVD in the French population, despite their consumption of a diet high in fat [107]. The French paradox has been attributed to the moderate intake of red wine, which is rich in polyphenols, such as resveratrol. However, clinical intervention studies of resveratrol in obese patients have yielded mixed results. Some studies have indicated reductions in fasting serum glucose, triglycerides, insulin, and homeostatic model assessment for insulin resistance (HOMA-IR), as well as several circulating markers of inflammation and intramyocellular lipid content [108, 109], but improvements in body weight and waist circumference are inconclusive [110, 111]. The variable responses to this polyphenol [110] likely reflect the influence of genetic variations on resveratrol metabolism and health effects. Along these lines, *SOD2* rs4880, which has been linked to obesity [112], is reported to influence the anti-inflammatory properties of resveratrol [113]. Another example is the *BDNF* rs6265 AA genotype encoding BDNF-Val66Met. BDNF-Met alleles are associated with increased C-reactive protein and energy intake in obese patients [114] and with overt thrombotic events in CVD patients [115]. Interestingly, BDNF-Met-expressing mice respond to the antithrombotic effects of resveratrol, whereas BDNF-Val-expressing mice do not [115]. Consequently, we propose that further investigations be conducted to explore the anti-obesity effects of resveratrol by focusing on relevant genetic profiles, including *SOD2* [113], *BDNF* [115], *PPAR*, *NR1I3* and *MTNR1B* polymorphisms (Fig. 1B).

## Genetically informed strategies for weight loss with specialized foods and supplements

Compliance with conventional weight-management programmes, including diet and exercise, can be challenging for many individuals, and this has led to a dramatic increase in overall use of slimming aids with various claims of effectiveness. Nonetheless, individual responses to supplements and nutraceuticals can vary, suggesting that genetic background may play a modifying role in individual responses [22].

For instance, coffee has become a widely favored beverage in recent years because of not only its caffeine content but also its rich array of bioactive compounds, such as polyphenols and chlorogenic acid, which have been associated with various health benefits [116]. In a study involving more than 15,000 women, regular coffee intake was proposed to modify genetic susceptibility to obesity [117]. This study relied on a GRS calculated from 77 BMI-associated loci and showed that women with a high obesity GRS who regularly consumed coffee showed promising trends toward improved body weight. We have also reported that a genetic variation in *CYP1A2* rs762551 enabling rapid caffeine metabolism modulates the relationship between appetite, BMI, and coffee intake, especially among those with genetic susceptibility to obesity [118]. These findings suggest that coffee consumption may contribute to counteracting the influence of genetic factors on weight-related outcomes, particularly among fast caffeine metabolizers [118].

In addition to coffee, green tea polyphenols, specifically catechins, have shown promise in promoting reductions in body weight and improvements in lipid profiles. The response to catechins is influenced by genetic variations, such as rs4680 (G/A), which affects the activity of catechol-*O*-methyltransferase (*COMT*), an enzyme involved in catechin metabolism. Diverse *COMT* genotypes among individuals may lead to varying reactions to nutraceuticals containing catechins [119].

Vitamin D has been linked to the regulation of insulin secretion and activity, countering the effects of certain obesity-related genetic factors, such as *FTO* risk alleles, especially among children [120]. These insights emphasize the important roles that both coffee and vitamin D can play in the management of body weight, as their effectiveness is influenced by an individual's genetic background.

Apples are renowned for being a rich source of polyphenols, dietary fiber, carotenoids, and various other essential nutrients, with polyphenols exerting antiobesity effects. These compounds are believed to achieve these benefits by scavenging free radicals and influencing signal transduction processes, particularly in adipose tissue [121]. A genetic influence is likely, as indicated by the reported interaction between the *IL6*–174 G/C polymorphism and the reduction in body fat induced by polyphenol-rich cloudy apple juice (CloA) [122]. Individuals with the C/C genotype experienced a substantial decrease in body fat after 4 weeks of CloA consumption [122].

## Genetic variations guiding lifestyle changes in weight management

Dietary habits, chrononutrition and PA choices have a genetic component [12], and genomic variations may thus inform lifestyle recommendations for body weight management.

Regular PA plays a vital role in preserving a healthy body weight, regulating body composition, and managing overall energy balance. Engaging in structured PA is linked to various health benefits, reducing the risk of chronic conditions. Even light-intensity PA can increase blood glucose levels, whereas prolonged sedentary behavior may have adverse effects [123], including overweight and obesity. Behavioral variations in physical activity and sedentary lifestyle are likely influenced by numerous factors, with emerging evidence suggesting genetic links and a bidirectional causal relationship between physical activity/sedentary behavior and adiposity-related traits. This intricate interplay may contribute to the development of a potentially vicious cycle [124].

Numerous studies indicate that individuals genetically predisposed to obesity may exhibit a diminished response to exercise. For instance, a study leveraging 21 identified BMI-associated SNPs demonstrated that individuals with a lower genetic risk of obesity showed more pronounced improvements in body weight, body fat, body fat percentage, and abdominal fat after a 1-year resistance exercise intervention, whereas the impact of exercise was less prominent among those at high genetic risk [125]. Similarly, earlier investigations into *FTO* single polymorphisms and exercise responses revealed that carriers of the risk alleles exhibit reduced responsiveness to exercise [126]. However, it is noteworthy that regular engagement in physical activity may contribute to mitigating the effect size of these polymorphisms associated with obesity. This underscores the significance of maintaining an active lifestyle, particularly in this specific, at-risk population [127–129].

Considering the intersection of genetics and lifestyle in influencing obesity, the choice of exercise emerges as a pivotal factor in weight management and overall health promotion. A published study involving 18,424 adults delved into the interplay between genetics and various exercise modalities in combating obesity. Among the exercises assessed, regular jogging stood out as the most effective at mitigating the genetic risk for obesity, as indicated by BMI, body fat percentage, and waist-to-hip ratio. Activities such as mountain climbing, walking, power walking, and extended yoga practices were also found to significantly reduce BMI in genetically predisposed individuals. Conversely, cycling, stretching exercises, swimming, and dance revolution did not counteract the genetic effects contributing to obesity [130]. This research provides valuable insights into personalized exercise recommendations for individuals with a genetic susceptibility to obesity, enhancing our understanding of the intricate interplay between genetics and PA in weight management. Our unpublished data indicate that adherence to different types of exercise has genetic influences that should be considered when enhancing the efficacy of PA for weight control.

Mis-timed eating is another lifestyle parameter that contributes to obesity [131, 132], likely by affecting eating behavior and appetite [133]. Genetic variation influences the impact of mistimed eating on adiposity, as indicated by a recent study that integrated data from more than 27,000 company employees. Individuals who were genetically predisposed to obesity (as calculated by a GRS incorporating 97 BMI variants) made poor-quality, calorie-dense food choices in larger quantities, especially in the workplace environment [134]. A significant association between late-night meal consumption, reduced overall weight loss and a slower weight loss rate has also been reported for individuals who are homozygous for the A allele of *PLIN1* (14995A/T), which encodes a circadian lipid droplet-stabilizing protein. Notably, food timing did not exert a comparable impact on weight loss among individuals with the T variant, further underscoring interindividual variability in weight loss outcomes influenced by genetics and meal timing [135].

Twin studies have also pointed to genetic influences on food timing, especially breakfast. In a study involving more than 200,000 participants, six genetic variants related to caffeine metabolism (*ARID3B*/*CYP1A1*), carbohydrate processing (*FGF21*), schizophrenia (*ZNF804A*), and circadian rhythm regulation (*METTL4*, *YWHAB*, and *YTHDF3*) were found to be associated with breakfast skipping [136]. Interestingly, the expression of the identified genes was enriched in the cerebellum. Of additional interest is the nature of the *METTL4*, *YWHAB*, and *YTHDF3* enzymes, which are important for N6-methyladenosine RNA transmethylation, underscoring the emerging need to further explore epitranscriptomics in metabolic disease [137]. Mendelian randomization experiments demonstrated causal links between genetically determined breakfast skipping and greater BMI [136]. Thus, eating the right time for some individuals may be more challenging but more crucial for their weight management and overall health.

## Conclusions and future directions

The rapid progress in the field of precision medicine largely stems from the appreciation of the key role of genetic variation in disease pathogenesis and response to treatment. Along the same lines, precision nutrition is expected to overcome current bottlenecks in the management of diet-related pathologies and become a ‘mainstay of medical care’ by the year 2030 [138].

By exploring the genomic perspective of personalized prevention and dietary management of obesity, this review has delved into the intricate interplay between genetics and dietary factors (Additional file 1: Table S1), particularly focusing on macronutrient tailoring. The sections addressing carbohydrate, lipid, protein, and fiber consumption underscore the importance of personalized approaches informed by genetic backgrounds. The identification of actionable genomic variants and the development of genetically informed strategies involving specialized foods and supplements offer promising avenues for more effective weight management.

Despite these advancements, bottlenecks in reducing the burden of obesity persist, emphasizing the need for a comprehensive understanding of the genetic variations guiding lifestyle changes in weight management. The intricate pathways and mechanisms related to actionable genomic variants provide a foundation for macronutrient stratification, paving the way for more targeted interventions.

When devising a diet plan, it is crucial to acknowledge that adjusting the proportion of one of the three macronutrients (carbohydrates, protein, or fat) will unavoidably influence the percentages of the other two unless there is a simultaneous modification in total caloric intake. However, the objective may not be to increase caloric intake, especially when the focus is on weight regulation. Specialized algorithms have been developed, including those of our team, to generate specific combinations of macronutrient percentages tailored for effective weight regulation by integrating genetic and other health parameters [7, 139, 140]. This approach, augmented by the capabilities of machine learning (ML) and artificial intelligence (AI), has the potential to offer personalized dietary advice by employing precision nutrition standards for the management of excessive body weight. Importantly, our exploration of the genetic variations guiding lifestyle changes extends beyond dietary considerations to encompass exercise and mistimed eating, acknowledging the multifaceted nature of personalized obesity management.

In the broader context, future research should focus on refining and expanding our understanding of the complex genetic landscape underlying obesity. Integrating data on actionable genomic variants with lifestyle recommendations and specialized interventions holds immense potential for enhancing the precision of personalized prevention and management strategies. Moreover, continued exploration of the dynamic interplay between genomics and obesity-related factors will contribute to the ongoing evolution of tailored approaches. As we advance, ongoing collaborative efforts and the integration of cutting-edge technologies will be essential to propel the field toward more effective, personalized solutions for obesity prevention and management.

In this era of advancing technologies, we have envisioned the future of precision nutrition through the use of “digital twin” models [141]. A digital twin represents a virtual replica of an individual, incorporating a comprehensive array of multimodal information, including genetics, microbiome composition, immune responses, metabolic activity, lifestyle factors, and anthropometric parameters. By continuously updating based on real-world data from these diverse sources, digital twins, enhanced by ML and AI algorithms, offer dynamic insights into an individual's response to dietary interventions. This advanced and integrated approach holds great promise in refining and optimizing personalized dietary recommendations, elevating the precision and efficacy of nutritional strategies for weight management and overall well-being [141].

### Supplementary Information

**Additional file 1**. **Table S1:** Published studies supporting the interplay between macronutrient intake, lifestyle and genetic background analyzed in this review article. **Table S2:** Actionable Gene List for Obesity Management (AnGeL) derived from 313 SNPs associated with macronutrient interactions in obesity management. **Table S3:** Enrichment results of AnGeL for REACTOME, WIKI and KEGG pathways related to Fig.1A, and for drugs predicted to be associated with the AnGeL gene set related to Fig. 1B. 

**Additional file 2. Figure S1**: Gene names associated with 313 SNPs reported to guide macronutrient intake (carbs, fat, protein and fiber; Additional file 1: Table S2) were analyzed against DISGENET, and the 15 most enriched disease ontologies were plotted to show their interconnections.

## Data Availability

Not applicable.

## Abbreviations

ADRB2: Adrenoceptor β 2
AI: Artificial intelligence
ALK: Anaplastic lymphoma kinase
APOA2: Apolipoprotein A-II
APOE4: Apolipoprotein E4
ARID3B: AT-rich interactive domain 3B
BDNF: Brain-derived neurotrophic factor
BMI: Body mass index
CloA: Cloudy apple juice
CNS: Central nervous system
CNV: Copy number variation
COMT: Catechol-*O*-methyltransferase
CYP1A2: Cytochrome P450 1A2
EIF2AK4: Eukaryotic translation initiation factor 2 alpha kinase 4
ERK: Extracellular signal-regulated kinase
FGF21: Fibroblast growth factor 21
FTO: Fat mass and obesity associated
GRS: Genetic risk score
GWASs: Genome-wide association studies
HOMA-IR: Homeostatic model assessment for insulin resistance
IL6: Interleukin 6
LBM: Lean body mass
LDL: Low-density lipoprotein
MAP2K5: Mitogen-activated protein kinase 5
MAP3: Mitogen-activated protein kinase 3
MAPK: Mitogen activated protein kinase
MC4R: Melanocortin 4 receptor
METTL4: N6-adenine-specific RNA methyltransferase 4
ML: Machine learning
MTNR1B: Melatonin receptor 1B
MUFAs: Monounsaturated fatty acids
NR1I3: Nuclear receptor subfamily 1 group I member 3
NTRK2: Neurotrophic receptor tyrosine kinase 2
PA: Physical activity
PLIN1: Perilipin 1
PPAR: Peroxisome proliferator-activated receptor
PRS: Polygenic risk score
PUFAs: Polyunsaturated fatty acids
RNA: Ribonucleic acid
SCFA: Short-chain fatty acid
SFA: Saturated fatty acid
SNP: Single nucleotide polymorphism
SOD2: Superoxide dismutase 2
STK33: Serine/threonine kinase 33
SYK: Spleen-associated tyrosine kinase
T2D: Type 2 diabetes
TCF7L2: Transcription factor 7 like 2
TFAP2B: Transcription factor AP-2 β
TPL2: Tumor progression locus 2
YWHAB: Tyrosine 3-monooxygenase/tryptophan 5-monooxygenase activation protein β
YTHDF3: YTH N6-methyladenosine RNA binding protein 3
ZNF804A: Zinc finger protein 804A

## References

[1] Loos RJF, Yeo GSH (2022). The genetics of obesity: from discovery to biology. Nat Rev Genet.

[2] Wardle J, Carnell S, Haworth CM, Plomin R (2008). Evidence for a strong genetic influence on childhood adiposity despite the force of the obesogenic environment. Am J Clin Nutr.

[3] Riveros-McKay F, Mistry V, Bounds R, Hendricks A, Keogh JM, Thomas H (2019). Genetic architecture of human thinness compared to severe obesity. PLoS Genet.

[4] Fehlert E, Wagner R, Ketterer C, Bohm A, Machann J, Fritsche L (2017). Genetic determination of body fat distribution and the attributive influence on metabolism. Obesity (Silver Spring).

[5] Graham SE, Clarke SL, Wu KH, Kanoni S, Zajac GJM, Ramdas S (2021). The power of genetic diversity in genome-wide association studies of lipids. Nature.

[6] Akbari P, Gilani A, Sosina O, Kosmicki JA, Khrimian L, Fang YY (2021). Sequencing of 640,000 exomes identifies GPR75 variants associated with protection from obesity. Science.

[7] Ramos-Lopez O, Cuervo M, Goni L, Milagro FI, Riezu-Boj JI, Martinez JA (2020). Modeling of an integrative prototype based on genetic, phenotypic, and environmental information for personalized prescription of energy-restricted diets in overweight/obese subjects. Am J Clin Nutr.

[8] Wang T, Heianza Y, Sun D, Huang T, Ma W, Rimm EB (2018). Improving adherence to healthy dietary patterns, genetic risk, and long term weight gain: gene-diet interaction analysis in two prospective cohort studies. BMJ.

[9] Khera AV, Chaffin M, Wade KH, Zahid S, Brancale J, Xia R (2019). Polygenic prediction of weight and obesity trajectories from birth to adulthood. Cell.

[10] Locke AE, Kahali B, Berndt SI, Justice AE, Pers TH, Day FR (2015). Genetic studies of body mass index yield new insights for obesity biology. Nature.

[11] Farooqi IS (2022). Monogenic obesity syndromes provide insights into the hypothalamic regulation of appetite and associated behaviors. Biol Psychiatry.

[12] Cole JB, Florez JC, Hirschhorn JN (2020). Comprehensive genomic analysis of dietary habits in UK Biobank identifies hundreds of genetic associations. Nat Commun.

[13] Grammatikopoulou MG, Skoufas E, Kanellakis S, Sanoudou D, Pavlopoulos GA, Eliopoulos AG (2023). Ageotypes revisited: the brain and central nervous system dysfunction as a major nutritional and lifestyle target for healthy aging. Maturitas.

[14] Salvestrini V, Sell C, Lorenzini A (2019). Obesity may accelerate the aging process. Front Endocrinol (Lausanne).

[15] Gkouskou K, Vasilogiannakopoulou T, Andreakos E, Davanos N, Gazouli M, Sanoudou D (2021). COVID-19 enters the expanding network of apolipoprotein E4-related pathologies. Redox Biol.

[16] Wadden TA, Tronieri JS, Butryn ML (2020). Lifestyle modification approaches for the treatment of obesity in adults. Am Psychol.

[17] European Commission Health Promotion and Disease Prevention Knowledge Gateway. 2021. https://knowledge4policy.ec.europa.eu/health-promotion-knowledge-gateway/dietary-fats-table-4_en

[18] Grammatikopoulou MG, Gkouskou KK, Gkiouras K, Bogdanos DP, Eliopoulos AG, Goulis DG (2022). The niche of n-of-1 trials in precision medicine for weight loss and obesity treatment: back to the future. Curr Nutr Rep.

[19] Sacks FM, Bray GA, Carey VJ, Smith SR, Ryan DH, Anton SD (2009). Comparison of weight-loss diets with different compositions of fat, protein, and carbohydrates. N Engl J Med.

[20] Chawla S, Tessarolo Silva F, Amaral Medeiros S, Mekary RA, Radenkovic D (2020). The effect of low-fat and low-carbohydrate diets on weight loss and lipid levels: a systematic review and meta-analysis. Nutrients.

[21] Lei L, Huang J, Zhang L, Hong Y, Hui S, Yang J (2022). Effects of low-carbohydrate diets versus low-fat diets on metabolic risk factors in overweight and obese adults: a meta-analysis of randomized controlled trials. Front Nutr.

[22] Gkouskou KK, Grammatikopoulou MG, Vlastos I, Sanoudou D, Eliopoulos AG (2021). Genotype-guided dietary supplementation in precision nutrition. Nutr Rev.

[23] Ludwig DS (2002). The glycemic index: physiological mechanisms relating to obesity, diabetes, and cardiovascular disease. JAMA.

[24] Templeman NM, Skovso S, Page MM, Lim GE, Johnson JD (2017). A causal role for hyperinsulinemia in obesity. J Endocrinol.

[25] Pawlak DB, Kushner JA, Ludwig DS (2004). Effects of dietary glycaemic index on adiposity, glucose homoeostasis, and plasma lipids in animals. Lancet.

[26] Ebbeling CB, Leidig MM, Feldman HA, Lovesky MM, Ludwig DS (2007). Effects of a low-glycemic load vs low-fat diet in obese young adults: a randomized trial. JAMA.

[27] Pittas AG, Das SK, Hajduk CL, Golden J, Saltzman E, Stark PC (2005). A low-glycemic load diet facilitates greater weight loss in overweight adults with high insulin secretion but not in overweight adults with low insulin secretion in the CALERIE trial. Diabetes Care.

[28] Astley CM, Todd JN, Salem RM, Vedantam S, Ebbeling CB, Huang PL (2018). Genetic evidence that carbohydrate-stimulated insulin secretion leads to obesity. Clin Chem.

[29] Qi Q, Chu AY, Kang JH, Jensen MK, Curhan GC, Pasquale LR (2012). Sugar-sweetened beverages and genetic risk of obesity. N Engl J Med.

[30] Brunkwall L, Chen Y, Hindy G, Rukh G, Ericson U, Barroso I (2016). Sugar-sweetened beverage consumption and genetic predisposition to obesity in 2 Swedish cohorts. Am J Clin Nutr.

[31] Haslam DE, McKeown NM, Herman MA, Lichtenstein AH, Dashti HS (2017). Interactions between genetics and sugar-sweetened beverage consumption on health outcomes: a review of gene-diet interaction studies. Front Endocrinol (Lausanne).

[32] Marti A, Corbalan MS, Martinez-Gonzalez MA, Forga L, Martinez JA (2002). CHO intake alters obesity risk associated with Pro12Ala polymorphism of PPARgamma gene. J Physiol Biochem.

[33] Martinez JA, Corbalan MS, Sanchez-Villegas A, Forga L, Marti A, Martinez-Gonzalez MA (2003). Obesity risk is associated with carbohydrate intake in women carrying the Gln27Glu beta2-adrenoceptor polymorphism. J Nutr.

[34] Cameron JD, Riou ME, Tesson F, Goldfield GS, Rabasa-Lhoret R, Brochu M (2013). The TaqIA RFLP is associated with attenuated intervention-induced body weight loss and increased carbohydrate intake in post-menopausal obese women. Appetite.

[35] Mandel AL, Breslin PA (2012). High endogenous salivary amylase activity is associated with improved glycemic homeostasis following starch ingestion in adults. J Nutr.

[36] Viljakainen H, Andersson-Assarsson JC, Armenio M, Pekkinen M, Pettersson M, Valta H (2015). Low copy number of the AMY1 locus is associated with early-onset female obesity in Finland. PLoS ONE.

[37] Mejia-Benitez MA, Bonnefond A, Yengo L, Huyvaert M, Dechaume A, Peralta-Romero J (2015). Beneficial effect of a high number of copies of salivary amylase AMY1 gene on obesity risk in Mexican children. Diabetologia.

[38] Falchi M, El-Sayed Moustafa JS, Takousis P, Pesce F, Bonnefond A, Andersson-Assarsson JC (2014). Low copy number of the salivary amylase gene predisposes to obesity. Nat Genet.

[39] Marcovecchio ML, Florio R, Verginelli F, De Lellis L, Capelli C, Verzilli D (2016). Low AMY1 gene copy number is associated with increased body mass index in prepubertal boys. PLoS ONE.

[40] Bonnefond A, Yengo L, Dechaume A, Canouil M, Castelain M, Roger E (2017). Relationship between salivary/pancreatic amylase and body mass index: a systems biology approach. BMC Med.

[41] Usher CL, Handsaker RE, Esko T, Tuke MA, Weedon MN, Hastie AR (2015). Structural forms of the human amylase locus and their relationships to SNPs, haplotypes and obesity. Nat Genet.

[42] Yong RY, Mustaffa SB, Wasan PS, Sheng L, Marshall CR, Scherer SW (2016). Complex copy number variation of AMY1 does not associate with obesity in two East Asian Cohorts. Hum Mutat.

[43] Rukh G, Ericson U, Andersson-Assarsson J, Orho-Melander M, Sonestedt E (2017). Dietary starch intake modifies the relation between copy number variation in the salivary amylase gene and BMI. Am J Clin Nutr.

[44] Heianza Y, Zhou T, Yuhang C, Huang T, Willett WC, Hu FB (2020). Starch digestion-related amylase genetic variants, diet, and changes in adiposity: analyses in prospective cohort studies and a randomized dietary intervention. Diabetes.

[45] Poole AC, Goodrich JK, Youngblut ND, Luque GG, Ruaud A, Sutter JL (2019). Human salivary amylase gene copy number impacts oral and gut microbiomes. Cell Host Microbe.

[46] Wang L, Wang H, Zhang B, Popkin BM, Du S (2020). Elevated fat intake increases body weight and the risk of overweight and obesity among Chinese adults: 1991–2015 trends. Nutrients.

[47] Astrup A, Grunwald GK, Melanson EL, Saris WH, Hill JO (2000). The role of low-fat diets in body weight control: a meta-analysis of ad libitum dietary intervention studies. Int J Obes Relat Metab Disord.

[48] U.S Department of Health and Human Services https://health.gov/our-work/nutrition-physical-activity/dietary-guidelines/previous-dietary-guidelines/2000. 2000. 10.3109/15360288.2015.1037530 26095483

[49] U.S Department of Health and Human Services https://health.gov/sites/default/files/2019-09/2015-2020_Dietary_Guidelines.pdf. 2015. 10.3109/15360288.2015.1037530 26095483

[50] Esposito K, Kastorini CM, Panagiotakos DB, Giugliano D (2011). Mediterranean diet and weight loss: meta-analysis of randomized controlled trials. Metab Syndr Relat Disord.

[51] Beulen Y, Martinez-Gonzalez MA, van de Rest O, Salas-Salvado J, Sorli JV, Gomez-Gracia E (2018). Quality of dietary fat intake and body weight and obesity in a Mediterranean population: secondary analyses within the PREDIMED trial. Nutrients.

[52] Casas-Agustench P, Arnett DK, Smith CE, Lai CQ, Parnell LD, Borecki IB (2014). Saturated fat intake modulates the association between an obesity genetic risk score and body mass index in two US populations. J Acad Nutr Diet.

[53] Celis-Morales CA, Lyall DM, Gray SR, Steell L, Anderson J, Iliodromiti S (2017). Dietary fat and total energy intake modifies the association of genetic profile risk score on obesity: evidence from 48 170 UK Biobank participants. Int J Obes (Lond).

[54] Huang T, Wang T, Heianza Y, Zheng Y, Sun D, Kang JH (2019). Habitual consumption of long-chain n-3 PUFAs and fish attenuates genetically associated long-term weight gain. Am J Clin Nutr.

[55] Li X, Zhou T, Ma H, Heianza Y, Champagne CM, Williamson DA (2020). Genetic variation in lean body mass, changes of appetite and weight loss in response to diet interventions: the POUNDS lost trial. Diabetes Obes Metab.

[56] Sanchez-Moreno C, Ordovas JM, Smith CE, Baraza JC, Lee YC, Garaulet M (2011). APOA5 gene variation interacts with dietary fat intake to modulate obesity and circulating triglycerides in a Mediterranean population. J Nutr.

[57] Stocks T, Angquist L, Banasik K, Harder MN, Taylor MA, Hager J (2012). TFAP2B influences the effect of dietary fat on weight loss under energy restriction. PLoS ONE.

[58] Goni L, Sun D, Heianza Y, Wang T, Huang T, Martinez JA (2019). A circadian rhythm-related MTNR1B genetic variant modulates the effect of weight-loss diets on changes in adiposity and body composition: the POUNDS lost trial. Eur J Nutr.

[59] Labayen I, Ruiz JR, Huybrechts I, Ortega FB, Arenaza L, Gonzalez-Gross M (2016). Dietary fat intake modifies the influence of the FTO rs9939609 polymorphism on adiposity in adolescents: the HELENA cross-sectional study. Nutr Metab Cardiovasc Dis.

[60] Mattei J, Qi Q, Hu FB, Sacks FM, Qi L (2012). TCF7L2 genetic variants modulate the effect of dietary fat intake on changes in body composition during a weight-loss intervention. Am J Clin Nutr.

[61] Qi Q, Bray GA, Hu FB, Sacks FM, Qi L (2012). Weight-loss diets modify glucose-dependent insulinotropic polypeptide receptor rs2287019 genotype effects on changes in body weight, fasting glucose, and insulin resistance: the preventing overweight using novel dietary strategies trial. Am J Clin Nutr.

[62] Grau K, Cauchi S, Holst C, Astrup A, Martinez JA, Saris WH (2010). TCF7L2 rs7903146-macronutrient interaction in obese individuals' responses to a 10-wk randomized hypoenergetic diet. Am J Clin Nutr.

[63] Garaulet M, Lee YC, Shen J, Parnell LD, Arnett DK, Tsai MY (2009). CLOCK genetic variation and metabolic syndrome risk: modulation by monounsaturated fatty acids. Am J Clin Nutr.

[64] Lin X, Qi Q, Zheng Y, Huang T, Lathrop M, Zelenika D (2015). Neuropeptide Y genotype, central obesity, and abdominal fat distribution: the POUNDS LOST trial. Am J Clin Nutr.

[65] Reddon H, Gueant JL, Meyre D (2016). The importance of gene-environment interactions in human obesity. Clin Sci (Lond).

[66] Smith CE, Tucker KL, Arnett DK, Noel SE, Corella D, Borecki IB (2013). Apolipoprotein A2 polymorphism interacts with intakes of dairy foods to influence body weight in 2 U.S. populations. J Nutr.

[67] Corella D, Tai ES, Sorli JV, Chew SK, Coltell O, Sotos-Prieto M (2011). Association between the APOA2 promoter polymorphism and body weight in Mediterranean and Asian populations: replication of a gene-saturated fat interaction. Int J Obes (Lond).

[68] Corella D, Arnett DK, Tsai MY, Kabagambe EK, Peacock JM, Hixson JE (2007). The -256T>C polymorphism in the apolipoprotein A-II gene promoter is associated with body mass index and food intake in the genetics of lipid lowering drugs and diet network study. Clin Chem.

[69] Lai CQ, Smith CE, Parnell LD, Lee YC, Corella D, Hopkins P (2018). Epigenomics and metabolomics reveal the mechanism of the APOA2-saturated fat intake interaction affecting obesity. Am J Clin Nutr.

[70] Vasilopoulou D, Markey O, Kliem KE, Fagan CC, Grandison AS, Humphries DJ (2020). Reformulation initiative for partial replacement of saturated with unsaturated fats in dairy foods attenuates the increase in LDL cholesterol and improves flow-mediated dilatation compared with conventional dairy: the randomized, controlled REplacement of SaturatEd fat in dairy on total cholesterol (RESET) study. Am J Clin Nutr.

[71] Pesta DH, Samuel VT (2014). A high-protein diet for reducing body fat: mechanisms and possible caveats. Nutr Metab (Lond).

[72] Leidy HJ, Clifton PM, Astrup A, Wycherley TP, Westerterp-Plantenga MS, Luscombe-Marsh ND (2015). The role of protein in weight loss and maintenance. Am J Clin Nutr.

[73] Gannon MC, Nuttall FQ (2010). Amino acid ingestion and glucose metabolism–a review. IUBMB Life.

[74] Kahleova H, Fleeman R, Hlozkova A, Holubkov R, Barnard ND (2018). A plant-based diet in overweight individuals in a 16-week randomized clinical trial: metabolic benefits of plant protein. Nutr Diabetes.

[75] McCarty MF (1999). Vegan proteins may reduce risk of cancer, obesity, and cardiovascular disease by promoting increased glucagon activity. Med Hypotheses.

[76] Goni L, Cuervo M, Milagro FI, Martinez JA (2015). A genetic risk tool for obesity predisposition assessment and personalized nutrition implementation based on macronutrient intake. Genes Nutr.

[77] Daily JW, Park S (2023). Association of plant-based and high-protein diets with a lower obesity risk defined by fat mass in middle-aged and elderly persons with a high genetic risk of obesity. Nutrients.

[78] Ramos-Lopez O, Riezu-Boj JI, Milagro FI, Cuervo M, Goni L, Martinez JA (2019). Models integrating genetic and lifestyle interactions on two adiposity phenotypes for personalized prescription of energy-restricted diets with different macronutrient distribution. Front Genet.

[79] de Luis DA, Aller R, Izaola O, Primo D, Urdiales S, Romero E (2015). Effects of a high-protein/low-carbohydrate diet versus a standard hypocaloric diet on weight and cardiovascular risk factors: role of a genetic variation in the rs9939609 FTO gene variant. J Nutrigenet Nutrigenomics.

[80] Merritt DC, Jamnik J, El-Sohemy A (2018). FTO genotype, dietary protein intake, and body weight in a multiethnic population of young adults: a cross-sectional study. Genes Nutr.

[81] Huang T, Qi Q, Li Y, Hu FB, Bray GA, Sacks FM (2014). FTO genotype, dietary protein, and change in appetite: the preventing overweight using novel dietary strategies trial. Am J Clin Nutr.

[82] Qi Q, Kilpelainen TO, Downer MK, Tanaka T, Smith CE, Sluijs I (2014). FTO genetic variants, dietary intake and body mass index: insights from 177,330 individuals. Hum Mol Genet.

[83] Qi Q, Downer MK, Kilpelainen TO, Taal HR, Barton SJ, Ntalla I (2015). Dietary intake, FTO genetic variants, and adiposity: a combined analysis of over 16,000 children and adolescents. Diabetes.

[84] Huang T, Zheng Y, Hruby A, Williamson DA, Bray GA, Shen Y (2017). Dietary protein modifies the effect of the MC4R genotype on 2-year changes in appetite and food craving: the POUNDS lost trial. J Nutr.

[85] Rukh G, Sonestedt E, Melander O, Hedblad B, Wirfalt E, Ericson U (2013). Genetic susceptibility to obesity and diet intakes: association and interaction analyses in the Malmo Diet and Cancer Study. Genes Nutr.

[86] Stocks T, Angquist L, Hager J, Charon C, Holst C, Martinez JA (2013). TFAP2B -dietary protein and glycemic index interactions and weight maintenance after weight loss in the DiOGenes trial. Hum Hered.

[87] Miketinas DC, Bray GA, Beyl RA, Ryan DH, Sacks FM, Champagne CM (2019). Fiber intake predicts weight loss and dietary adherence in adults consuming calorie-restricted diets: the POUNDS lost (preventing overweight using novel dietary strategies) study. J Nutr.

[88] Bozzetto L, Costabile G, Della Pepa G, Ciciola P, Vetrani C, Vitale M (2018). Dietary fibre as a unifying remedy for the whole spectrum of obesity-associated cardiovascular risk. Nutrients.

[89] Grube B, Chong PW, Lau KZ, Orzechowski HD (2013). A natural fiber complex reduces body weight in the overweight and obese: a double-blind, randomized, placebo-controlled study. Obesity (Silver Spring).

[90] Huls A, Wright MN, Bogl LH, Kaprio J, Lissner L, Molnar D (2021). Polygenic risk for obesity and its interaction with lifestyle and sociodemographic factors in European children and adolescents. Int J Obes (Lond).

[91] Enoksson S, Talbot M, Rife F, Tamborlane WV, Sherwin RS, Caprio S (2000). Impaired in vivo stimulation of lipolysis in adipose tissue by selective beta2-adrenergic agonist in obese adolescent girls. Diabetes.

[92] Bachman ES, Dhillon H, Zhang CY, Cinti S, Bianco AC, Kobilka BK (2002). betaAR signaling required for diet-induced thermogenesis and obesity resistance. Science.

[93] Tan PY, Mitra SR (2020). The combined effect of polygenic risk from FTO and ADRB2 gene variants, odds of obesity, and post-hipcref diet differences. Lifestyle Genom.

[94] Hosseini-Esfahani F, Koochakpoor G, Daneshpour MS, Mirmiran P, Sedaghati-Khayat B, Azizi F (2017). The interaction of fat mass and obesity associated gene polymorphisms and dietary fiber intake in relation to obesity phenotypes. Sci Rep.

[95] Veronese N, Solmi M, Caruso MG, Giannelli G, Osella AR, Evangelou E (2018). Dietary fiber and health outcomes: an umbrella review of systematic reviews and meta-analyses. Am J Clin Nutr.

[96] Partula V, Deschasaux M, Druesne-Pecollo N, Latino-Martel P, Desmetz E, Chazelas E (2020). Associations between consumption of dietary fibers and the risk of cardiovascular diseases, cancers, type 2 diabetes, and mortality in the prospective NutriNet-Sante cohort. Am J Clin Nutr.

[97] Lyssenko V, Lupi R, Marchetti P, Del Guerra S, Orho-Melander M, Almgren P (2007). Mechanisms by which common variants in the TCF7L2 gene increase risk of type 2 diabetes. J Clin Invest.

[98] Zeggini E, Scott LJ, Saxena R, Voight BF, Marchini JL, Hu T (2008). Meta-analysis of genome-wide association data and large-scale replication identifies additional susceptibility loci for type 2 diabetes. Nat Genet.

[99] Hindy G, Sonestedt E, Ericson U, Jing XJ, Zhou Y, Hansson O (2012). Role of TCF7L2 risk variant and dietary fibre intake on incident type 2 diabetes. Diabetologia.

[100] Heni M, Herzberg-Schafer S, Machicao F, Haring HU, Fritsche A (2012). Dietary fiber intake modulates the association between variants in TCF7L2 and weight loss during a lifestyle intervention. Diabetes Care.

[101] Thanati F, Karatzas E, Baltoumas FA, Stravopodis DJ, Eliopoulos AG, Pavlopoulos GA (2021). FLAME: a web tool for functional and literature enrichment analysis of multiple gene lists. Biology (Basel).

[102] Wang J, Vasaikar S, Shi Z, Greer M, Zhang B (2017). WebGestalt 2017: a more comprehensive, powerful, flexible and interactive gene set enrichment analysis toolkit. Nucleic Acids Res.

[103] Eliopoulos AG, Das S, Tsichlis PN (2006). The tyrosine kinase Syk regulates TPL2 activation signals. J Biol Chem.

[104] Gkirtzimanaki K, Gkouskou KK, Oleksiewicz U, Nikolaidis G, Vyrla D, Liontos M (2013). TPL2 kinase is a suppressor of lung carcinogenesis. Proc Natl Acad Sci.

[105] Gong J, Fang C, Zhang P, Wang PX, Qiu Y, Shen LJ (2019). Tumor progression locus 2 in hepatocytes potentiates both liver and systemic metabolic disorders in mice. Hepatology.

[106] Colonna L, Catalano G, Chew C, D'Agati V, Thomas JW, Wong FS (2010). Therapeutic targeting of Syk in autoimmune diabetes. J Immunol.

[107] Renaud S, de Lorgeril M (1992). Wine, alcohol, platelets, and the French paradox for coronary heart disease. Lancet.

[108] Walker JM, Eckardt P, Aleman JO, da Rosa JC, Liang Y, Iizumi T (2019). The effects of trans-resveratrol on insulin resistance, inflammation, and microbiota in men with the metabolic syndrome: a pilot randomized, placebo-controlled clinical trial. J Clin Transl Res.

[109] Timmers S, Konings E, Bilet L, Houtkooper RH, van de Weijer T, Goossens GH (2011). Calorie restriction-like effects of 30 days of resveratrol supplementation on energy metabolism and metabolic profile in obese humans. Cell Metab.

[110] Mongioi LM, La Vignera S, Cannarella R, Cimino L, Compagnone M, Condorelli RA (2021). The role of resveratrol administration in human obesity. Int J Mol Sci.

[111] Hillsley A, Chin V, Li A, McLachlan CS (2022). Resveratrol for weight loss in obesity: an assessment of randomized control trial designs in ClinicalTrials.gov. Nutrients.

[112] Lewandowski L, Kepinska M, Milnerowicz H (2020). Alterations in concentration/activity of superoxide dismutases in context of obesity and selected single nucleotide polymorphisms in genes: SOD1, SOD2, SOD3. Int J Mol Sci.

[113] Capeleto D, Barbisan F, Azzolin V, Dornelles EB, Rogalski F, Teixeira CF (2015). The anti-inflammatory effects of resveratrol on human peripheral blood mononuclear cells are influenced by a superoxide dismutase 2 gene polymorphism. Biogerontology.

[114] Goldfield GS, Walsh J, Sigal RJ, Kenny GP, Hadjiyannakis S, De Lisio M (2021). Associations of the BDNF Val66Met polymorphism with body composition, cardiometabolic risk factors, and energy intake in youth with obesity: findings from the HEARTY study. Front Neurosci.

[115] Amadio P, Colombo GI, Tarantino E, Gianellini S, Ieraci A, Brioschi M (2017). BDNFVal66met polymorphism: a potential bridge between depression and thrombosis. Eur Heart J.

[116] Lee A, Lim W, Kim S, Khil H, Cheon E, An S (2019). Coffee intake and obesity: a meta-analysis. Nutrients.

[117] Wang T, Huang T, Kang JH, Zheng Y, Jensen MK, Wiggs JL (2017). Habitual coffee consumption and genetic predisposition to obesity: gene-diet interaction analyses in three US prospective studies. BMC Med.

[118] Gkouskou KG, Georgiopoulos G, Vlastos I, Lazou E, Chaniotis D, Papaioannou TG (2022). CYP1A2 polymorphisms modify the association of habitual coffee consumption with appetite, macronutrient intake, and body mass index: results from an observational cohort and a cross-over randomized study. Int J Obes (Lond).

[119] Hursel R, Janssens PL, Bouwman FG, Mariman EC, Westerterp-Plantenga MS (2014). The role of catechol-O-methyl transferase Val(108/158)Met polymorphism (rs4680) in the effect of green tea on resting energy expenditure and fat oxidation: a pilot study. PLoS ONE.

[120] Lourenco BH, Qi L, Willett WC, Cardoso MA, Team AS (2014). FTO genotype, vitamin D status, and weight gain during childhood. Diabetes.

[121] Vallee Marcotte B, Verheyde M, Pomerleau S, Doyen A, Couillard C (2022). Health benefits of apple juice consumption: a review of interventional trials on humans. Nutrients.

[122] Barth SW, Koch TC, Watzl B, Dietrich H, Will F, Bub A (2012). Moderate effects of apple juice consumption on obesity-related markers in obese men: impact of diet-gene interaction on body fat content. Eur J Nutr.

[123] Del Pozo-Cruz J, Garcia-Hermoso A, Alfonso-Rosa RM, Alvarez-Barbosa F, Owen N, Chastin S (2018). Replacing sedentary time: meta-analysis of objective-assessment studies. Am J Prev Med.

[124] Schnurr TM, Stallknecht BM, Sorensen TIA, Kilpelainen TO, Hansen T (2021). Evidence for shared genetics between physical activity, sedentary behaviour and adiposity-related traits. Obes Rev.

[125] Klimentidis YC, Bea JW, Lohman T, Hsieh PS, Going S, Chen Z (2015). High genetic risk individuals benefit less from resistance exercise intervention. Int J Obes (Lond).

[126] Rankinen T, Rice T, Teran-Garcia M, Rao DC, Bouchard C (2010). FTO genotype is associated with exercise training-induced changes in body composition. Obesity (Silver Spring).

[127] Kilpelainen TO, Qi L, Brage S, Sharp SJ, Sonestedt E, Demerath E (2011). Physical activity attenuates the influence of FTO variants on obesity risk: a meta-analysis of 218,166 adults and 19,268 children. PLoS Med.

[128] Li S, Zhao JH, Luan J, Ekelund U, Luben RN, Khaw KT (2010). Physical activity attenuates the genetic predisposition to obesity in 20,000 men and women from EPIC-Norfolk prospective population study. PLoS Med.

[129] Vimaleswaran KS, Li S, Zhao JH, Luan J, Bingham SA, Khaw KT (2009). Physical activity attenuates the body mass index-increasing influence of genetic variation in the FTO gene. Am J Clin Nutr.

[130] Lin WY, Chan CC, Liu YL, Yang AC, Tsai SJ, Kuo PH (2019). Performing different kinds of physical exercise differentially attenuates the genetic effects on obesity measures: evidence from 18,424 Taiwan Biobank participants. PLoS Genet.

[131] Garaulet M, Gomez-Abellan P, Alburquerque-Bejar JJ, Lee YC, Ordovas JM, Scheer FA (2013). Timing of food intake predicts weight loss effectiveness. Int J Obes (Lond).

[132] Jakubowicz D, Barnea M, Wainstein J, Froy O (2013). High caloric intake at breakfast vs. dinner differentially influences weight loss of overweight and obese women. Obesity (Silver Spring).

[133] Ruddick-Collins LC, Morgan PJ, Fyfe CL, Filipe JAN, Horgan GW, Westerterp KR (2022). Timing of daily calorie loading affects appetite and hunger responses without changes in energy metabolism in healthy subjects with obesity. Cell Metab.

[134] Dashti HS, Hivert MF, Levy DE, McCurley JL, Saxena R, Thorndike AN (2020). Polygenic risk score for obesity and the quality, quantity, and timing of workplace food purchases: a secondary analysis from the ChooseWell 365 randomized trial. PLoS Med.

[135] Garaulet M, Vera B, Bonnet-Rubio G, Gomez-Abellan P, Lee YC, Ordovas JM (2016). Lunch eating predicts weight-loss effectiveness in carriers of the common allele at PERILIPIN1: the ONTIME (Obesity, Nutrigenetics, Timing, Mediterranean) study. Am J Clin Nutr.

[136] Dashti HS, Merino J, Lane JM, Song Y, Smith CE, Tanaka T (2019). Genome-wide association study of breakfast skipping links clock regulation with food timing. Am J Clin Nutr.

[137] Sanoudou D, Gkouskou KK, Eliopoulos AG, Mantzoros CS (2022). Epitranscriptomic challenges and promises in metabolic diseases. Metabolism.

[138] Rodgers GP, Collins FS (2020). Precision nutrition—the answer to “what to eat to stay healthy”. JAMA.

[139] Cuevas-Sierra A, Milagro FI, Guruceaga E, Cuervo M, Goni L, Garcia-Granero M (2022). A weight-loss model based on baseline microbiota and genetic scores for selection of dietary treatments in overweight and obese population. Clin Nutr.

[140] Gkouskou K, Lazou E, Skoufas E, Eliopoulos AG (2021). Genetically guided mediterranean diet for the personalized nutritional management of type 2 diabetes mellitus. Nutrients.

[141] Gkouskou K, Vlastos I, Karkalousos P, Chaniotis D, Sanoudou D, Eliopoulos AG (2020). The “virtual digital twins” concept in precision nutrition. Adv Nutr.

