# Supplementary figures and images for: A genomics perspective of personalized prevention and management of obesity
Source: Hum Genomics. 2024 Jan 29;18:4. doi: 10.1186/s40246-024-00570-3 (PMC10823690; doi:10.1186/s40246-024-00570-3)

## Slide 1
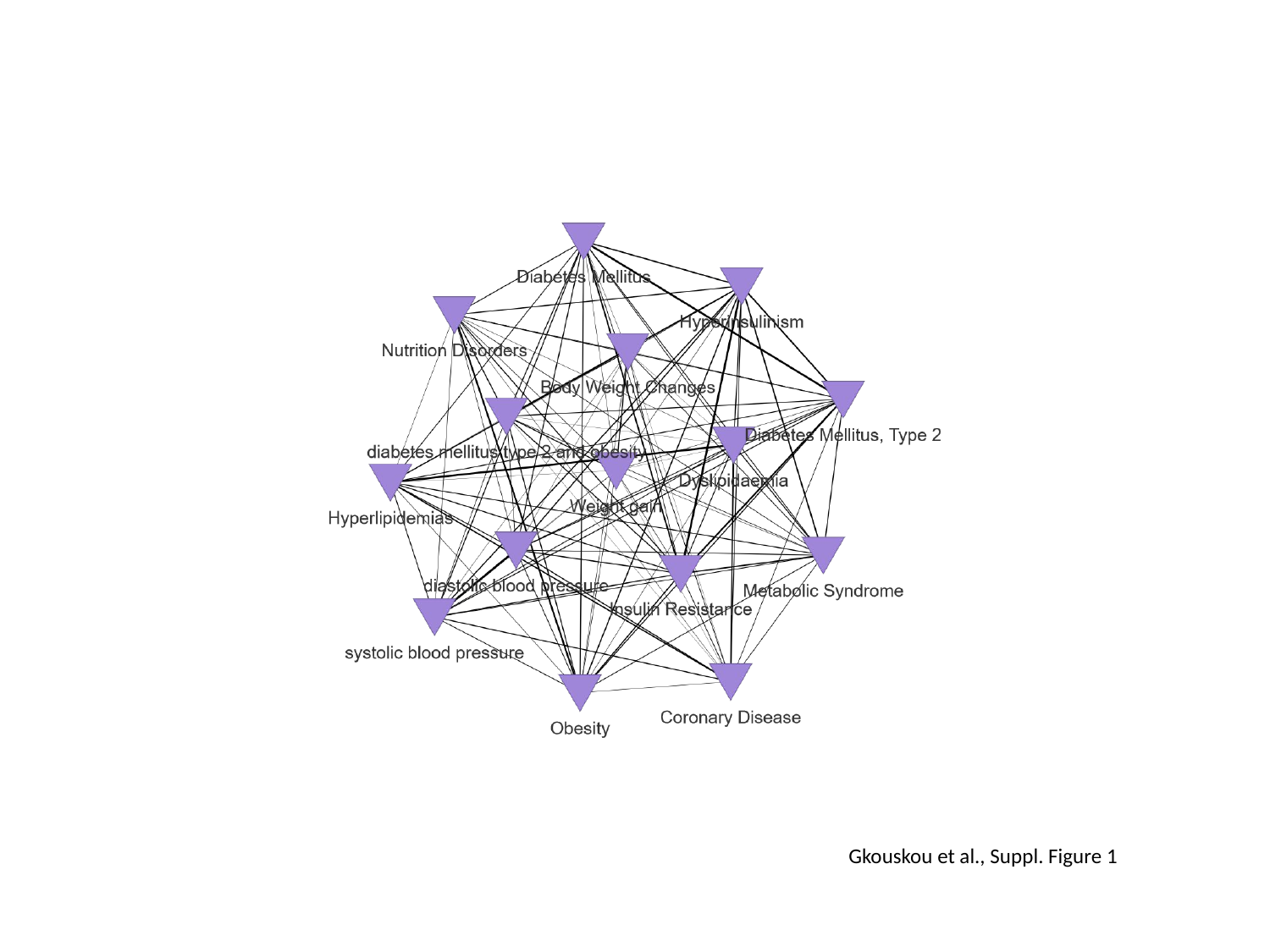

Gkouskou et al., Suppl. Figure 1

Supplement: Supplementary file 2 — Additional file 2. Figure S1: Gene names associated with 313 SNPs reported to guide macronutrient intake (carbs, fat, protein and fiber; Additional file 1: Table S2) were analyzed against DISGENET, and the 15 most enriched disease ontologies were plotted to show their interconnections. [file 40246_2024_570_MOESM2_ESM.pptx]
